# PTEN: a new dawn in Parkinson’s disease treatment

**DOI:** 10.3389/fncel.2025.1497555

**Published:** 2025-03-10

**Authors:** Xinghuang Yang, Tianqi Liu, Hong Cheng

**Affiliations:** ^1^Medical College, Yangzhou University, Yangzhou, China; ^2^Jiangsu Key Laboratory of Experimental & Translational Non-Coding RNA Research, Yangzhou University Medical College, Institute of Translational Medicine, Yangzhou University, Yangzhou, China

**Keywords:** PTEN, Parkinson’s disease, clinical therapeutics, pathology, signaling pathway

## Abstract

In recent years, the study of phosphatase and tension homolog (PTEN) has gradually become a research hotspot. As an important oncogene, the role of PTEN in cancer has long been widely recognized and intensively studied, but it has been relatively less studied in other diseases. Parkinson’s disease (PD) is a neurodegenerative refractory disease commonly observed in middle-aged and elderly individuals. The etiology and pathogenesis of PD are numerous, complex, and incompletely understood. With the continuous deepening of research, numerous studies have proven that PTEN is related to the occurrence of PD. In this review, we discuss the relationship between PTEN and PD through the phosphorylation and ubiquitination of PTEN and other possible regulatory mechanisms, including the role of RNA molecules, exosomes, transcriptional regulation, chemical modification, and subtype variation, with the aim of clarifying the regulatory role of PTEN in PD and better elucidating its pathogenesis. Finally, we summarize the shortcomings of PTEN in PD research and highlight the great potential of its future application in PD clinical treatment. These findings provide research ideas and new perspectives for the possible use of PTEN as a PD therapeutic target for targeted drug development and clinical application in the future.

## 1 Introduction

### 1.1 PTEN: background, structure, functions, diseases

#### 1.1.1 Background

Phosphatase and tensin homolog (PTEN), also known as MMAC, was discovered in 1997 as a candidate tumor suppressor gene on chromosome 10q23 (Li et al., 1997). In addition to its role as an important common tumor suppressor controlling cell survival, proliferation, migration, and metabolism, it also functions to regulate homeostasis within normal cells and, in particular, controls several important cell signaling pathways, such as the PI3K/AKT pathway, the adhesion plaque kinase/p130Cas pathway, and the Shc/Raf/ERK1/2 signaling cascade, as identified by Maehama and Dixon (1998, 1999), Tamura et al. (1998), Chetram and Hinton (2012), respectively. The PI3K/AKT/mTor pathway is mostly associated with Parkinson’s disease (PD). Aberrant PTEN expression may affect this signaling pathway, leading to disrupted proliferation or death of neuronal cells, resulting in PD. The identified PTEN family genes include *PTEN*, *TPTE*, *TPIP*, and *Ci-vsp*. In addition to *PTEN*, three genes, *TPTE*, *TPIP*, and *Ci-vsp*, have not yet been confirmed to have a significant correlation with PD (Kurokawa et al., 2012) and will not be repeated in this review. PTENα (PTEN-Long) and PTENβ are the currently describable PTEN isoforms. PTENα can co-localize with typical PTEN to promote the activation of mitochondria-targeted kinase and energy production (Liang et al., 2014), which is associated with PD.

#### 1.1.2 Structure

**FIGURE 1:**
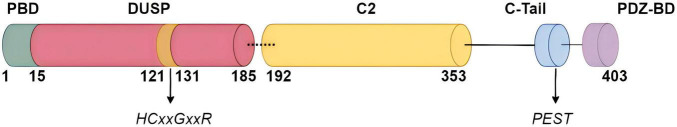
The structure of phosphatase and tension homolog (PTEN). PTEN consists of phosphatidylinositol [PI(4,5)P2]-binding sequence (PBD), dual-specificity phosphatase structural domain (DUSP) and C2 domains, followed by the C-Tail and the PDZ-BD. The DUSP sequence contains the characteristic motif HCxxGxxR, and the tail of the C-tail contains the PEST sequence. Where PBD is green, DUSP is red, C2 is yellow, PDZ-BD2 is purple, HCxxGxxR is orange, and PEST is blue. Created with Figdraw (www.figdraw.com) (ID: TYWUT5ff20).

Human PTEN, a member of the classical protein tyrosine phosphatase gene (PTPase) superfamily, encodes a PTEN protein containing 403 amino acids. The basic structure of the protein consists of a conserved catalytic structural domain flanked by non-catalytic sequences and regulatory regions (Figure 1): the N-terminal phosphatidylinositol [PI(4,5)P2]-binding sequence (PBD), the dual-specificity phosphatase structural domain (DUSP), the C2 structural domain, the carboxyl-terminal tail structural domain (C-Tail/CTT), and the PDZ-structural domain binding sequence (PDZ-BD) (Masson and Williams, 2020).

The PBD sequence (residues 1–14) determines how PTEN binds to anionic lipids and upregulates phosphatase activity through denaturation (Campbell et al., 2003). The DUSP sequence (residues 15–185) is the catalytic core of PTEN and carries the PTP signature motif C(x)5 R (HCxxGxxR) in certain regions. The DUSP sequence functions to promote phosphatidylinositol substrate binding and dephosphorylation, which is closely related to the PI3k/AKT pathway (Chow and Baker, 2006). The C2 structural domain (residues 192–353) serves as the site of action of the phosphatase, with which it shares an extensive interface. The C2 structural domain is responsible for recruiting PTEN to the membrane and efficiently localizing the catalytic structural domain to the cell membrane (Lee et al., 1999). The C-Tail (residues 353–403) is disordered and absent from crystals with a recognizable PEST sequence (Pro, Glu, Ser, Thr) in its tail, which acts as a marker for protein hydrolysis. Its absence reduces PTEN expression in cells. It has also been demonstrated that this sequence is non-essential for the ubiquitination of PTEN by the NEDD4 E3 ubiquitin-protein ligase (Wang K. et al., 2022). PDZ-BD (Thr-Val-Lys) is a target of many proteins with scaffolding functions and plays an important role in regulating PTEN stability and mediating protein–protein interactions (Adey et al., 2000).

#### 1.1.3 Functions

Phosphatase and tension homolog is dependent on both phosphatase and lipid phosphatase activities. In addition, it has a non-phosphatase-dependent “scaffolding” function (Lee et al., 2018).

Initially, PTEN was identified as a protein phosphatase that was shown to contribute to tumor suppression (Li et al., 2019). Previously, it was discovered that PTEN showed intrinsic phosphatase activity *in vitro* on peptides phosphorylated at Tyr, Ser, and Thr sites (Myers et al., 1997). By contrast, recent studies have shown that its activity is regulated by the carboxy-terminal tail (CTT) and exhibits inhibition (Smith et al., 2022). Subsequently, since the main substrate of PTEN is a lipid membrane component, it mostly functions as a lipid phosphatase with classical biological functions. In its unphosphorylated state (Masson et al., 2016), PTEN dephosphorylates three sites on the phosphatidylinositol-(3,4,5)-triphosphate (PIP3) ring to generate PIP2, which restricts Akt recruitment and activation, thereby affecting the entire PI3k/AKT pathway. In addition to its phosphatase activity, PTEN can participate in ubiquitination regulation; i.e., E3 ubiquitin ligases containing the WW region can inhibit PTEN function by blocking PTEN dimerization and membrane recruitment (Lee Y. R. et al., 2019).

Phosphatase and tension homolog often acts as a scaffolding protein between the nucleus and cytoplasm, independently exerting its non-phosphatase-dependent function. In the nucleus, PTEN regulates cell proliferation and transcription while maintaining genome stability. In the cytoplasm, PTEN mainly regulates apoptosis. The transport function of PTEN in the nucleus is likely involved in the pathogenesis of many diseases, including PD. On the basis of the transport function, oxidative, acetylation, SUMO modification, protein interactions, and transcriptional, miRNA, and methylation regulation have been discovered in PTEN. Ho et al. (2020) recent study on the nuclear function of PTEN revealed how PTEN is transported, retained, or cleared from the nucleus. Another study showed that several lysine residues play an important role in the nuclear translocation mechanism, laying the groundwork for subsequent research (Salmena and Pandolfi, 2007).

#### 1.1.4 Diseases

In recent years, PTEN has played an important role in various diseases, including cancer-related disorders characterized by uncontrolled cellular reproduction. Among them, Cowden syndrome, also called PTEN missense tumor syndrome (PHTS), is caused by germline PTEN mutations and was the first classical syndrome to be identified. Many studies have attempted to enhance surveillance and prevention by profiling its clinical manifestations (Yehia et al., 2020). Since PHTS increases the risk of concurrent cancers in tissues of various body systems, studies on PTEN have expanded to include related disorders of the thyroid, breast, endometrium, and kidneys. For example, it has been found that PTEN deficiency promotes the metastasis of cholangiocarcinoma (CCA) (Jiang et al., 2023). In addition, neurodevelopmental disorders, including autism spectrum disorders (ASD), are more likely to result in multifactorial disorders with dysregulation of other known ASD susceptibility genes under PTEN-deficient conditions (Rademacher et al., 2025).

The protective effect of PTEN on neuronal cells has been demonstrated. PTEN reduces peripheral nerve injury by modulating synaptic regeneration, inflammatory and immune responses, neuronal apoptosis, neurotrophic-related factors, and related signaling pathways, such as PI3K/AKT/mTOR, although its effects in the central nerve are poorly explained. Based on the fact that PTEN may have similar protective or restorative effects on neurons, an increasing number of studies have found new evidence that PTEN regulates PD. For example, Johnson et al. (2023) found that PTEN can act as an upstream activator of Fyn kinase, leading to the death of dopaminergic neurons in PD. Since PTEN has been effective in tumor therapy, it makes practical sense to use PTEN as a target to treat PD. In tumors, PTEN often regulates cancer cell proliferation by inhibiting the PI3K/AKT/mTOR and Wnt pathways (Chaudagar et al., 2023a,b). Therefore, using PTEN as a target may similarly regulate the disordered proliferative homeostasis of neuronal cells, thereby reducing the death of damaged neurons and the occurrence of PD.

### 1.2 Parkinson’s disease (PD)

Parkinson’s disease is the second most common neurodegenerative disease in the world, with a global prevalence of more than 6 million people, representing a 2.5-fold increase over the prevalence in the previous generation (Dorsey et al., 2018; GBD 2016 Neurology Collaborators., 2019). According to statistics, the incidence of PD in China is increasing, with the number of new patients approaching 100,000 per year. The incidence rate of PD increases with age, and approximately 1% of the world’s elderly (> 60 years) are affected (Tysnes and Storstein, 2017). Among the risk factors for PD, the most important is the age factor. This is followed by genetic factors, with more than 100 genetic risk loci having been identified and characterized (Blauwendraat et al., 2020), while a GWAS has identified nearly 80 different susceptibility loci (Nalls et al., 2019). In addition to these, environmental and behavioral factors have been found to play a role in the pathogenesis of PD (Blauwendraat et al., 2020), primarily associated with pesticide/herbicide exposure, military-related chemicals, and head strikes (Payami et al., 2023). Typical clinical manifestations of PD include motor and non-motor symptoms, including resting tremors, bradykinesia, and postural and gait disturbances, while non-motor symptoms include cognitive and psychiatric abnormalities, sleep disturbances, autonomic dysfunction, and sensory disturbances. These symptoms not only reduce the patient’s quality of life but also represent a burden to the family and society (Foltynie et al., 2024).

The predominant pathological change in PD is the degenerative death of dopaminergic neurons in the substantia nigra of the midbrain, which leads to a significant reduction in dopamine (DA) content in the striatum (caudate nucleus plus pallidum), which impairs its signaling function and thus causes the disease. The pathological markers of PD include α-synuclein (α-syn) and iron loading in the substantia nigra. α-syn is a soluble protein expressed presynaptically and perinuclearly in the central nervous system, which aggregates to form typical inclusion bodies called Lewy bodies. By contrast, iron loading is closely related to the ferroptosis response and is an important mechanism of PD dopaminergic neuronal death (Wang Z. L. et al., 2022).

Despite unprecedented progress in research on the pathogenesis of PD, its etiology remains full of enigmas, and there is currently no effective method for its treatment and prevention, which remain considerable challenges. Combined with extensive experiments, genetic analyses, and pathological autopsies, the possible pathogenesis of PD now focuses on biological themes such as neurosynapses, lysosomes, mitochondria, oxidative stress, ferroptosis, protein aggregation, immune-inflammatory responses, and intestinal ecological dysregulation (Dong-Chen et al., 2023; Ye et al., 2023). Among them, mitochondrial dysfunction, oxidative stress, ferroptosis, and other mechanisms may have a strong relationship with PTEN, which will be covered in detail in this article. In addition, PTEN has a strong genetic association with PD. In the latest PD-associated genetic test (PDGT) (Westenberger et al., 2024) and an observational study called PD GENEration (Cook et al., 2024), among pathogenic genes (e.g., SNCA, LRRK2, VPS35, LRP10, PRKN, PINK1, DJ1), PTEN-induced kinase1 (PINK1 – the classic single gene for PD) remained a high-risk factor for triggering PD. However, there is insufficient information on the correlation between PTEN and factors such as environmental and lifestyle behaviors that trigger PD, which needs to be explored urgently.

## 2 Regulation of PTEN phosphorylation in PD

As the main regulator of the PI3K/AKT/mTOR (PAM) pathway, PTEN plays a role as a lipid phosphatase dephosphorylating PIP3 to PIP2, thus blocking the pathway of AKT activation by PI3K. Studies have also shown that the PAM pathway can inhibit ferroptosis, which is the key “trigger switch” of PD, thereby playing an indirect protective role in PD. Reducing PTEN overexpression or inducing PTEN mutation may increase the degree of inhibition of ferroptosis by the PAM pathway, thus achieving protection from PD.

### 2.1 Ferroptosis

Ferroptosis is a novel iron-dependent mode of programmed cell death that occurs in two main ways. The first pathway is a non-enzymatic pathway called the classical Fenton reaction, in which divalent iron reacts with hydrogen peroxide to produce trivalent iron and toxic free radicals. The second pathway is an enzymatic pathway that functions through the action of lipoxygenase (LOX) and cytochrome P450 oxidoreductase (POR). These two pathways continuously generate chain reactions that ultimately allow the oxidation of polysaturated fatty acids (PL-PUFA) in the cell membrane to generate toxic lipid peroxides (PUFA-OOH) (Shen et al., 2018; Dong et al., 2019), which manifest as an accumulation of iron and lipid peroxidation. Interestingly, these toxic lipid peroxides can be reduced back to PL-PUFA by glutathione peroxidase 4 (GPX4). GPX4 functions mainly in the System Xc system (Gao M. et al., 2019), which can be indirectly activated by glutathione (GSH) and is required for the degradation of lipid peroxides (Liu et al., 2022). Recently, Sun et al. (2023) showed that dopaquinone (DAQ) can directly bind to and modify the cysteine residues of GPX4, leading to the loss of dopaminergic neurons in PD. GSH can directly supply cysteine residues to DAQ, and the process of ubiquitination after DAQ modification is mediated by the ubiquitin ligase NEDD4 (Sun et al., 2023), thus refining the mechanism of ferroptosis.

As an iron-dependent chemical response, ferroptosis is closely linked to the transportation and absorption of iron. Abnormalities in serum transferrin (Tf) and its receptor TfR1, such as palmitoylation, have been found to cause neurodegeneration (Park and Chung, 2019). In addition, the overexpression of divalent metal ion transporter 1 (DMT1), an important iron uptake protein, contributes to iron accumulation in the substantia nigra and the loss of dopaminergic neurons (Zhang et al., 2017). Ferroptosis has received increasing attention in recent years. For example, in basic research, Ryan et al., identified iron-loaded microglia as playing a key role in neurodegenerative diseases such as PD (Kenkhuis et al., 2023). In clinical studies, Xia et al. (2021) found that the use of iron-metal grafts in orthopedic surgery should be considered a risk factor for developing PD, adding to the possibility of ferroptosis as a cause of PD. Wang Z. L. et al. (2022) concluded that ferroptosis is likely an important mechanism of dopaminergic neuronal death in PD, as well as a potential target for intervention and treatment.

Recent studies have suggested that PTEN directly regulates ferroptosis. Kezhuo et al. (2022) found that embryonic fibroblasts of PTEN-knockout mice were less sensitive to ferroptosis induced by the ferroptosis inducer Erastin, verifying that PTEN plays a facilitating role in ferroptosis. Glutamate-cysteine ligase modifier subunit (GCLM), which encodes a glutamate and cysteine ligase modifier (Lu, 2009), can inhibit ferroptosis by promoting GSH synthesis (Stockwell et al., 2017). Therefore, when GCLM was knocked down in PTEN-knockout cells, the sensitivity of the cells to Erastin was increased, confirming that the deletion of PTEN could upregulate the expression of GCLM. Therefore, PTEN may inhibit the expression of GCLM, thereby accelerating ferroptosis (Kezhuo et al., 2022). Ferroptosis may be an important reason for PD; however, whether PTEN knockdown or mutation can effectively inhibit the occurrence of ferroptosis and protect against PD remains to be further investigated.

### 2.2 PAM pathway

**FIGURE 2:**
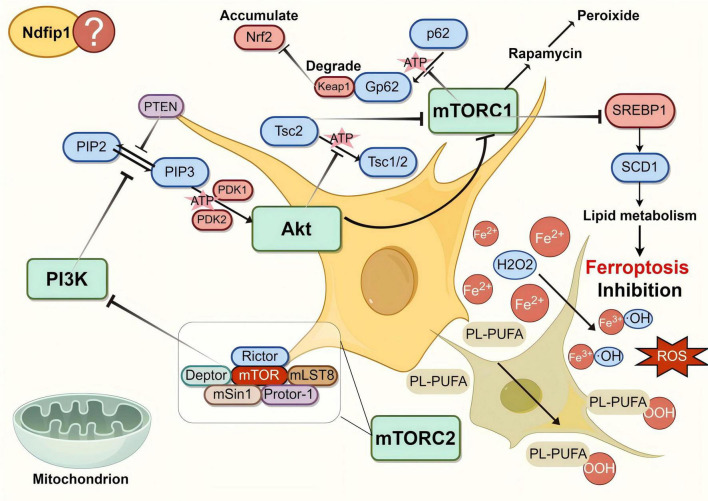
The diagram of the PI3K/AKT/mTOR (PAM) signaling pathway. The PAM pathway consists of three basic components, namely PI3 kinase (PI3K), AKT and mammalian target of rapamycin-like protein (mTOR), as well as various signaling molecules in the bypass pathway, which can inhibit ferroptosis. Phosphatase and tension homolog (PTEN), as a lipid phosphatase, dephosphorylates PIP3 to generate PIP2, which restricts the recruitment and activation of AKT, thus affecting the whole signaling pathway. Ndfip1 can also participate in the pathway, though the specific mechanism is unknown. Created with Figdraw (www.figdraw.com) (ID: ATWSU9f94b).

The PAM pathway is mainly composed of three basic components: PI3 kinase (PI3K), protein kinase B (PKB/AKT), and mammalian target of rapamycin-like protein (mTOR) (Figure 2). PI3K belongs to the family of lipid kinases, and full activation of PI3K (class I phosphorylate) converts PIP2 to PIP3, which leads to the recruitment of the serine/threonine kinases AKT and PDK1 near the intracellular membrane. At the same time, the AKT serine 308 site is phosphorylated (Hong-Ju et al., 2010). mTOR is a serine/threonine protein kinase that exists as two complexes, mTORC1 and mTORC2 (Ting and Fu-Sheng, 2016). Activated AKT can trigger mTOR, which interacts with other protein molecules and performs different functions (Li et al., 2022). As a classical oncogenic signaling pathway, dysregulation of the PAM pathway promotes cancer development (Ong et al., 2016; Lee J. H. et al., 2019). For example, in a PI3K-activated xenograft mouse model of breast cancer, inhibition of both ferroptosis and mTORC1 resulted in almost complete tumor regression (Cheng et al., 2022).

Activation of the PAM pathway protects against PD, in which PTEN plays an important role. A recent study by Junmei Y et al., found that sterol regulatory element-binding protein 1 (SREBP1), as a mTORC1-dependent protein regulated by the SREBP1-transcriptional target stearoyl cofactor desaturase-1 (SCD1), can regulate lipid metabolism and exert a ferroptosis-inhibitory effect (Yi et al., 2020), which may protect against PD. The dephosphorylation of PIP3 by PTEN prevents the activation of AKT by PI3K and inhibits the activation of the PAM signaling pathway, reducing its inhibitory effect on ferroptosis, which leads to PD. Activation of the PAM signaling pathway also regulates bypass signaling axes such as Nrf2/HO-1, PKD1, and BDNF/CREB, exerting a protective effect against PD (Gao et al., 2022). Indeed, it was found that rosmarinic acid (RA) promotes antioxidant gene expression by regulating the AKT/Nrf2 signaling pathway to achieve neuroprotective effects in a 1-methyl-4-phenyl-1,2,3,6-tetrahydropyridine (MPTP)-induced zebrafish PD model, while RA can simultaneously inhibit PTEN expression (Zhao Y. et al., 2020). PTEN-induced kinase 1 (PINK1) was also found to stimulate the physiological effects of neuronal development by activating the PKA-CREB-BDNF signaling axis in a feed-forward loop (Soman et al., 2021).

## 3 Regulation of PTEN ubiquitination in PD

**FIGURE 3:**
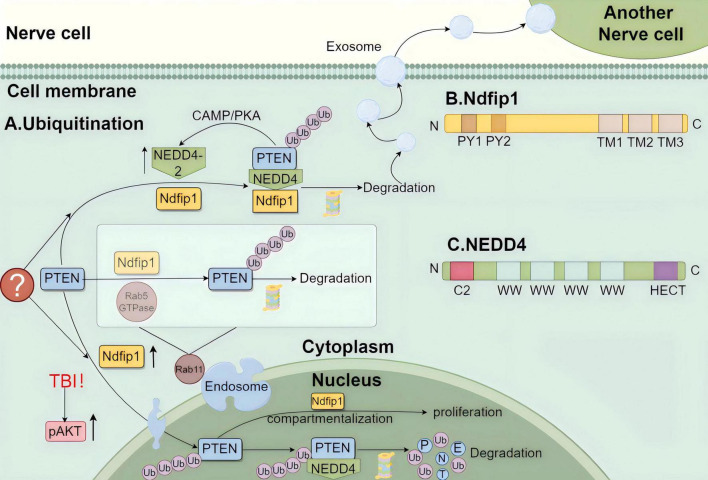
The diagram of the regulatory mechanism of ubiquitination consisting of phosphatase and tension homolog (PTEN) with Ndfip1 and NEDD4. **(A)** The regulation of ubiquitination in neurons by PTEN. Before PTEN enters the nucleus, it is ubiquitinated by Rab5 GTPase and Ndfip1 in the cytoplasmic endosomes containing Rab11, and PTEN can also bind to Ndfip1 and promote NEDD4 to complete its ubiquitination, and the binding of Ndfip1 to NEDD4-2 is increased. The increased abundance of Ndfip1 promoted the entry of PTEN into the nucleus, especially after traumatic brain injury (TBI). After, PTEN entered the nucleus, it could also ubiquitinate by binding to NEDD4, and the compartmentalization of PTEN in the nucleus was regulated by Ndfip1. The degraded PTEN after ubiquitination can be transported by exosomes to other neuronal cells to function. **(B)** Illustration of the gene structure of Ndfip1:Ndfip1 consists of two PY modules at the N-terminus: PY1, PY2 and three transmembrane hydrophobic regions at the C-terminus: TM1, TM2, TM3. **(C)** Illustration of the gene structure of NEDD4: NEDD4 consists of the N-terminal C2 structural domain, the C-terminal HECT structural domain, and the 2–4 WW structural domains in the middle. Created with Figdraw (www.figdraw.com) (ID: IIPAY1fafa).

Activation of the PAM pathway plays a protective role in PD, and PTEN is the most critical link in the activation of AKT by PI3K. If PTEN is degraded, then AKT is likely to be activated by PI3K, maintaining the highly protective effect of the PAM pathway on PD (Tan et al., 2020). Ubiquitination, which is mediated by various ubiquitinating and de-ubiquitinating enzymes, is an important mechanism for PTEN degradation. The nervous system contains many ubiquitin ligases, among which the E3 ubiquitin ligase NEDD4 is representative (Figure 3) and closely associated with Ndfip1 and PTEN. NEDD4, Ndfip1, and PTEN form a complex ubiquitination regulatory network, which will be highlighted later in the article, while the other enzymes will not.

### 3.1 Ndfip1-NEDD4 system

#### 3.1.1 Ndfip1

The Ndfip1 (Nedd4 family interacting protein 1) protein, named for its specific binding to the NEDD4 protein family (Jolliffe et al., 2000), is inextricably linked to NEDD4 and is a potential target for the ubiquitination of NEDD4 family proteins. The important structural domains of Ndfip1 are two PY modules at the N-terminal end, namely PY1 and PY2, and three transmembrane hydrophobic regions at the C-terminal end, namely TM1, TM2, and TM3. *In vivo*, Ndfip mediates ubiquitination through the combination of the PY modules (most of them PY1) and the WW domains of NEDD4 proteins and has several sub-subunit functions. Binding to NEDD4 proteins can mediate ubiquitination, thereby affecting subcellular localization and cell function (Harvey et al., 2002).

Recent studies have found that Ndfip1 is highly expressed in the adult brain, including the right and left hemispheres, pituitary gland, and thalamus, suggesting that its protective effect on neuronal cell specificity is related to its protective effect against PD. At the cellular level, Ndfip1 protects neurons in PD models. For example, Hammond et al. (2014) found that Ndfip1 maintains synapse number and neuronal integrity in neocortical neurons during development. Similar protective effects were demonstrated in both 1-methyl-4-phenylpyridinium (MPP +) and rotenone-induced PD cell models (Jiashun et al., 2013; Liu et al., 2020). At the molecular level, Ndfip1 is mainly able to reduce iron loading. Indeed, Howitt found that elevated levels of Ndfip1 in neurons were associated with upregulated iron levels in the substantia nigra of patients with PD (Howitt et al., 2014). Traeger found that the ubiquitin-activating enzyme E1 UBA-6 and the bridging protein Ndfip1 can interact, and Ndfip1 deficiency reduces the degradation of human membrane ferroportin (FPN) (Traeger et al., 2022). Jia et al. (2015) found that Ndfip1 attenuated 6-OHDA-induced iron loading by regulating the degradation of DMT1. Ubiquitination of Ndfip1 with DMT1 in the Ndfip1/NEDD4 system is involved in the neurocellular protection pathway against high levels of metal toxicity (Howitt et al., 2009).

The normal function of PTEN cannot be achieved without the help of Ndfip1. Under homeostasis, PTEN is distributed in the cytoplasm of cerebral cortex neurons, whereas under cerebral ischemia, PTEN is transferred from the cytoplasm to the nucleus to accomplish nuclear localization. The nuclear accumulation of PTEN is closely related to the upregulation of Ndfip1 (Howitt et al., 2012). It was later demonstrated that traumatic brain injury (TBI), as a pathophysiological stimulus, promotes PTEN nuclear translocation in neurons, which is dependent on an increase in Ndfip1 abundance (Goh et al., 2014). Before PTEN enters the nucleus, Rab5 GTPase acts in conjunction with Ndfip1 to ubiquitinate PTEN. PTEN ubiquitination is localized to Rab11-containing endosomes in the cytoplasm that mediate PTEN nuclear translocation and exosomal secretion (Li et al., 2014). Following the entry of PTEN into the nucleolus, Ndfip1 can regulate cell proliferation by controlling the nuclear compartmentalization of PTEN (Howitt et al., 2015). Both Ndfip1 and Ndfip2 are involved in the regulation of PTEN/AKT (Mund and Pelham, 2010). When TBI occurs, the elevation of pAKT is a consequence of the decrease in PTEN in the cytoplasm due to the entry of PTEN into the nucleus, which leads to a decrease in the interfering effect of PTEN on the activation of AKT by PI3K and the derepression of pAKT (Goh et al., 2014). Ndfip1 is involved in the entire process of transport, degradation, and signaling activation of PTEN in neurons.

#### 3.1.2 NEDD4

Ubiquitination is mediated by three main enzymes, namely ubiquitin-activating enzyme E1, ubiquitin-conjugating enzyme E2, and ubiquitin ligase E3, all of which play important roles in determining substrate specificity (Weissman, 2001). The NEDD4 protein is a member of the HECT family of ubiquitin ligases E3. Its basic structure consists of an N-terminal C2 structural domain, a C-terminal HECT structural domain, and 2–4 WW structural domains in the middle. The WW structural domains can bind to certain proteins, such as the PY module of Ndfip1. However, binding to PTEN can only be realized through the HECT structural domains and the C2 structural domains of the NEDD4 protein, which are relatively independent of each other (Fouladkou et al., 2008; Wang et al., 2008).

In the nervous system, PTEN is closely related to NEDD4. Kwak et al. (2010) identified NEDD4 as the E3 ligase of PTEN in neurons and proposed that the ubiquitination of NEDD4 plays an important role in regulating the level and activity of PTEN in the PI3K/AKT pathway. Whether *in vivo* or *in vitro*, both PTEN and NEDD4 are localized in sensory neurons (Christie et al., 2012), and NEDD4 is responsible for mediating the ubiquitination of PTEN within neurons. Several lines of evidence suggest that the NEDD4-PTEN pathway is involved in many physiopathological processes and that NEDD4 is a target downstream of PTEN-dependent signaling (Ahn et al., 2008). By contrast, (Hsia et al. (2014) found a feedback loop mechanism between NEDD4 and PTEN, which suggests that PTEN can upregulate NEDD4 levels via the CAMP/PKA pathway (Yang et al., 2008). In other words, NEDD4 acts not as a substrate but as an upstream regulator of PTEN, thereby promoting neuronal survival. These findings further clarify the relationship between NEDD4 and PTEN.

Studies have shown that NEDD4 promotes neuronal growth by degrading PTEN (Christie et al., 2012). Since Ndfip1 drives PTEN into the nucleus, the binding of PTEN to Ndfip1 promotes its ubiquitination of PTEN by NEDD4, especially after TBI, which contributes to neuronal survival (Howitt et al., 2012). This process was validated by Goh et al. (2014) using a mouse trauma model. Interestingly, Ndfip1 is also able to bind to NEDD4, which catalyzes the degradation of ubiquitin-bound target proteins, including PTEN. During this process of ubiquitination, the binding of Ndfip1 to NEDD4-2 increases, while the binding of Ndfip1 to NEDD4-1 does not (Traeger et al., 2022). That is, PTEN can either be ubiquitinated by entering the nucleus with the help of Ndfip1 or degraded prematurely by ubiquitination errors mediated by the Ndfip1 in the complex with NEDD4-2. How this complex mechanism affects the entire process of PTEN degradation by NEDD4, whether neurons continue to survive and grow, and whether it is protective against PD remains to be investigated.

### 3.2 Other enzymes mediating ubiquitination and deubiquitination

In addition to the classical Ndfip1-NEDD4 system, many enzymes can mediate PTEN ubiquitination, including WWP1/2, Smurf1/2, CHIP, XIAP, TRIM27/RFP, SPOP, and MKRN-1. The ubiquitination activity of WWP2 on unphosphorylated PTEN even exceeds that of NEDD4 (Chen et al., 2016), while WWP1 inhibits the dimerization and membrane recruitment function of PTEN by polyubiquitination (Lee Y. R. et al., 2019). In terms of sites of action, few studies related to ubiquitination enzymes other than the WWP family have been reported in the nervous system and brain tissue. In terms of disease studies, except for NEDD4, more studies have focused on the tumor inhibitory effects of PTEN, whereas there is almost a gap in the studies on PD.

In addition to ubiquitination, de-ubiquitination, which is a reverse process of ubiquitination, helps maintain protein stability and functionality. Enzymes that mediate PTEN de-ubiquitination include ubiquitin-specific proteases (USP), ovarian tumor proteases (OTU), and so on. The main members of the USP family are USP7 (HAUSP), USP11, USP13, and USP18, whereas the OTU family includes OTUD3. For example, it was found that USP11, which is regulated by FOXO (Park et al., 2019), can act as a regulator of the PTEN/PI3K pathway by deubiquitinating PTEN, thereby enhancing its stability and indirectly inhibiting PI3K signaling (Park et al., 2019). It is presumable that the process continues to inhibit downstream AKT and mTOR signaling and attenuates the inhibition of ferroptosis by the PAM pathway, thereby inducing PD.

Despite progress in this field, there is still vast room for exploration regarding the impact of PTEN on PD occurrence after its involvement in the regulation of ubiquitination.

## 4 Other possible mechanisms of PTEN regulation in PD

**FIGURE 4:**
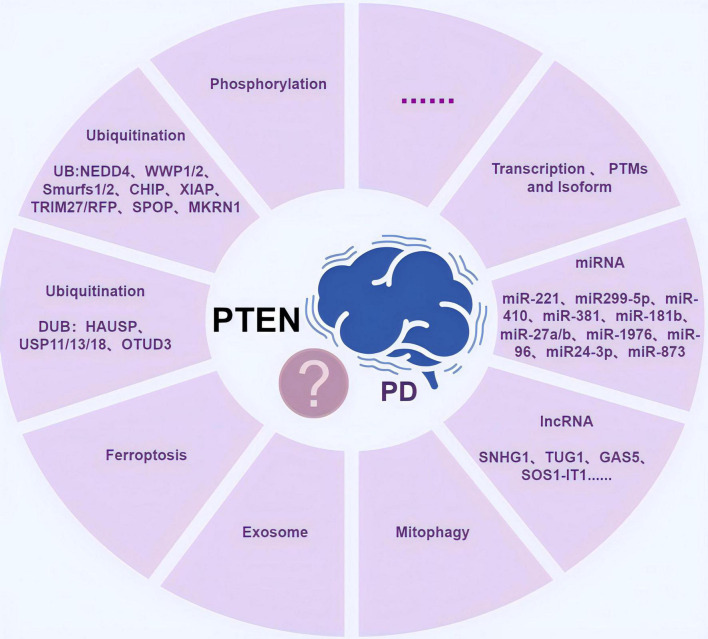
The diagram of possible mechanisms by which phosphatase and tension homolog (PTEN) regulates Parkinson’s disease (PD). It includes phosphorylation, ubiquitination (including ubiquitinating enzymes and deubiquitinating enzymes), ferroptosis, exosome, mitophagy, RNAs (miRNAs and lncRNAs), transcription, post-translational chemical modifications (PTMs), and different variant isoforms (PTENα and PTENβ), as well as other undiscovered possible mechanisms. Created with Figdraw (www.figdraw.com) (ID: AYPOI0900b).

In addition to regulating phosphorylation and ubiquitination, PTEN may regulate PD via other mechanisms (Figure 4), including mitochondrial autophagy and exocytosis.

### 4.1 PTEN and mitophagy

Mitochondria provide energy for normal neuronal function; therefore, stable mitochondrial function is especially important for nerve cell survival (Devine and Kittler, 2018). Mitochondrial autophagy is a pathway by which cells selectively encapsulate damaged mitochondria and transport them to the lysosome for degradation, thereby maintaining mitochondrial integrity (Lu et al., 2023). Growing evidence suggests that mitochondrial autophagy plays an important role in neurodegenerative diseases such as PD (Barazzuol et al., 2020). Among these, mitochondrial dysfunction (González-Rodríguez et al., 2021) and impaired mitochondrial autophagy (Quinn et al., 2020) are the two main factors contributing to PD. Mitochondrial quality control is an important mechanism that ensures that mitochondrial autophagy can be maintained (Pickles et al., 2018). The human PTEN-inducible putative kinase 1 (PINK1) and Parkin/PRKN are associated with mitochondrial quality control and are a source of regional vulnerability in PD (Ge et al., 2020), of which Parkin is the major causative gene for autosomal recessive juvenile parkinsonism (AR-JP). Mutations in both PINK1 and PRKN can trigger hereditary PD *in vivo* and *in vitro* via mitochondrial autophagy (Valente et al., 2004; Han et al., 2023). A recent study found that the combination of heterozygous PRKN and homozygous PTEN-induced PINK1 mutations resulted in earlier PD onset (Song et al., 2023). To address this issue, another study identified a small molecule that activates the PRKN mutant, which cannot be phosphorylated by PINK1, causing the cell to resume mitochondrial autophagy (Sauvé and Gehring, 2024).

Currently, PINK1 and Parkin are the major pathways that mediate mitochondrial autophagy (Harper et al., 2018). Phosphorylated kinase PINK1 recruits PRKN to damaged mitochondria and activates it. PRKN acts as an E3 ubiquitin ligase that ubiquitinates substrates on the surface of mitochondria, degrading them via proteasomes or lysosomes. Promoting the normal progression of dysfunctional mitochondrial autophagy reduces the occurrence of neurodegenerative diseases such as PD. Thus, activating positive regulators such as PINK1 and Parkin or inhibiting negative regulators such as phosphatases and DUBs appears to be necessary to restore impaired mitochondrial autophagy (Li et al., 2023).

Multiple studies have shown that mitochondrial autophagy mechanisms are not consistent across cells. Macroscopically, studies have refined the secondary mechanism by which PINK1 activates PRKN via anterograde feedback, which is responsible for approximately one-quarter of mitochondrial autophagy processes in cells (Fakih et al., 2023). Locally, it has been found that the mitochondrial outer membrane protein Synaptojanin2 (SYNJ2) can transport PINK1 mRNA through an RNA-binding structural domain, thereby supplying distal localized mitochondrial autophagy (Harbauer et al., 2022). To better understand and control the extent of mitochondrial autophagy in cells, many studies have focused on the PINK1–PRKN signaling axis. Because the physiological level of phosphorylated Ub (p-S65-Ub) used to modify mitochondria is very low, one study monitored PINK1–PRKN signaling in tissue cells by developing and characterizing a series of novel p-S65-Ub antibodies (Watzlawik et al., 2024b). Studies have also determined the basal and maximal activation of PINK1 and PRKN using rodent specimens and cells from patients with PD (Watzlawik et al., 2024a), clearly exploring the upper and lower boundaries of the degree of mitochondrial autophagic activity.

In addition, mitochondrial autophagy has been found to be mediated by a variety of substances or accompanied by altered levels of numerous factors, providing a direction for in-depth studies of mitochondrial autophagy. It has been found that mitochondrial autophagy is inhibited in patients with PD with mutations in the GBA (glucosylceramidase beta) gene, which is accompanied by decreased levels of AMBRA1 (Autophagy and beclin 1 regulator 1) (Di Rienzo et al., 2024), although the mechanism is unclear. It has been found that BNIP3L (BCL2/adenovirus E1B interacting protein 3-like) mediates mitochondrial autophagy and thus stimulates mitochondrial remodeling in optic nerve oligodendrocytes (Yazdankhah et al., 2021), providing ideas for the study of PD in the future. Chimeras composed of a mitochondria-targeting sequence (MTS) and ATG16L1-binding peptide (ABP) or LC3-interacting (LIR) have been shown to target and promote mitochondrial autophagy to ameliorate mitochondrial dysfunction in a PD cell model (Mei et al., 2023).

### 4.2 PTEN and RNA molecules

Ribonucleic acid (RNA) is a carrier of genetic information in biological cells. Compared with DNA, RNA has a small molecular weight and highly variable content and has been a hot research topic in the last few years. RNAs are categorized into messenger RNAs and non-coding RNAs according to their structure and function, and non-coding RNAs are divided by size. To date, the known RNAs associated with PTEN and PD are mainly non-coding RNAs, including miRNAs and long-stranded non-coding RNAs (lncRNAs). The main function of miRNAs is to inhibit the expression of target genes and play a role in gene disruption, whereas lncRNAs mainly regulate the transcription, translation, and chromosomal modification of genes (Shaath et al., 2022).

#### 4.2.1 miRNA

MicroRNAs (miRNAs) are endogenous non-coding single-stranded small RNA molecules, 19–25 nucleotides in length, which bind to the 3’-untranslated regions of their target genes to block mRNA translation or trigger mRNA degradation, thereby inhibiting gene expression at the post-transcriptional level (He et al., 2014). Previous studies have demonstrated the role of miRNA-mediated regulation of gene expression in disease progression (Preethi et al., 2022). PTEN is a target gene of miRNAs, and the interaction between them has been extensively studied in various cancers, including the role of PTENP1, the pseudogene of PTEN, and miR-20a in breast cancer (Gao X. et al., 2019). While the study of PTEN and miRNAs in PD is still in its infancy, it is considered a valuable field of research to investigate their relationship and protective effects in PD.

Many miRNAs are involved in PD development and are related to the proliferation, apoptosis, and differentiation of dopaminergic neurons, but the exact mechanism is still unclear (Cerri and Blandini, 2019). However, with the deepening of research, many researchers have found that miRNAs can regulate PD through PTEN. Indeed, miR-221 has been shown to directly target PTEN to eliminate its protective effect on pAKT expression in PC12 cells. In addition, miR-221 has moderate predictive ability for PD and is very likely to become a clinical biomarker for PD in the future (Zamanian et al., 2024). Yejun et al. (2023) found that miR-299-5p targeted SP3 to inhibit apoptosis in a PD cell model via the PTEN/AKT/mTOR pathway. Ge et al. (2019) found that miR-410 exerted neuroprotective effects on 6-OHDA-induced PD cell models by regulating the PTEN/AKT/mTOR signaling axis. Guo et al. (2023) found that miR-381 binds to EGR1 and upregulates PTEN/P53 to alleviate PD. miRNAs can directly or indirectly target PTEN. Overexpression of miRNAs can decrease PTEN expression, thus acting on the signaling axis where PTEN is located, ultimately regulating the protective effect on neuronal cells in PD models. It is worth noting that miRNAs have an antagonistic association with PTEN, and PTEN overexpression can also attenuate the effects of miRNAs.

Since abnormal autophagy levels associated with neuronal damage are observed in many patients with PD and animal models (Wang et al., 2016), many studies have focused on miRNA regulation of cellular autophagy. In the PAM pathway, mTORC1 is known to bind to the ULK complex and inhibit cellular autophagy; thus, PTEN is often combined with the PAM pathway in the study of PD (Zeng et al., 2015). Li et al. (2018) reported that miR-181b overexpression functioned to suppress cellular autophagy and reduce cellular damage in PD through the PTEN/AKT/mTOR pathway. Many researchers have since made similar findings, with Zhou et al. (2021) revealing that miR-221 had the same effect on 6-OHDA-induced PC12 cells.

In addition to common cellular autophagy, mitochondrial autophagy has emerged as a central mechanism in the pathogenesis of PD in recent years; thus, miRNAs are frequently studied in combination with mitochondrial autophagy. For example, Novak et al. (2022) demonstrated that mutations in PINK1 are strongly associated with the onset of PD using midbrain dopamine (mDA) neurons differentiated from human induced pluripotent stem cells with the ILE368ASN mutation in the PINK1 gene. Kim et al. (2016) demonstrated that miR-27a/b inhibited the autophagic clearance of damaged mitochondria by lysosomes through the translational repression of PINK1. Moreover, Qiu et al. (2023) found that overexpression of miR-1976 targets PINK1 to increase apoptosis and mitochondrial damage in dopaminergic neurons, thereby increasing the risk of PD and thus may be a biomarker for PD. Wang et al. (2021) found that pramipexole attenuated MPP + -induced cellular and MPTP-induced neuronal damage in mice by directly reducing miR-96 activation of bnip3-mediated mitochondrial autophagy. A subsequent study also demonstrated that increased reactive oxygen species in mitochondria strongly oxidized PTEN to inactivate it, thereby enhancing the signaling activity of the PAM pathway (Kim et al., 2018), perfecting the mechanism by which mitochondrial autophagy triggers PD.

MicroRNAs have several roles and are linked to many molecules, providing additional considerations for future research. For example, circEPS15 can rescue dopamine (DA) neuronal degeneration through the miR-24-3p/PINK1 axis-mediated improvement of mitochondrial functions (Zhou et al., 2023). Another study revealed that Ndfip1 is a direct target of miR-873, which depends on the NDFIP1/AKT/mTOR axis to exert inhibitory effects on hepatocellular carcinoma (Yuyu et al., 2019). Whether miR-873 has the same biological effect in PD models deserves to be further explored.

#### 4.2.2 lncRNA

In addition to miRNAs, many lncRNAs, a class of highly heterogeneous RNAs consisting of more than 200 nucleotides in length, are also involved in the regulation of PD. A significant proportion of lncRNAs have now been identified as important regulatory molecules in neurodegenerative diseases, including PD (Wan et al., 2017).

These lncRNAs include small molecule host gene 1 (SNHG1), taurine upregulated gene 1 (TUG1), and Growth Arrest Specific 5 (GAS5). SNHG1 can target miR-153-3p to stimulate PTEN/AKT/mTOR signaling and induce neurotoxicity in SH-SY5Y cells (Zhao J. et al., 2020). TUG1 also promotes PD by regulating the miR-152-3p/PTEN pathway (Zhai et al., 2020). GAS5 binds to miR-150 and targets Fosl1, thereby activating the PTEN/AKT/mTOR pathway, which promotes apoptosis, inhibits neuronal activity, and accelerates PD progression (Ma et al., 2022). SOS1-IT1 reduces damage to MPP+ -induced SK-N-SH cells via miR-124-3p, an effect that is reversed by the overexpression of PTEN, a target of miR-124-3p, while the SOS1-IT1/miR-124-3p/PTEN axis also modulates the activity of the AKT/mTOR pathway (Fan et al., 2022). Similar to miRNAs, all of these lncRNAs can also target PTEN to exert neuroprotective effects with the help of the signaling axis where PTEN is located, mainly the PI3K/AKT/mTOR axis. Interestingly, for some miRNAs and lncRNAs, the overexpression of PTEN has the potential to attenuate or even reverse this neuroprotective effect.

### 4.3 Transcriptional regulation of PTEN

In addition to regulation by RNAs, PTEN transcription is also regulated by several transcription factors. Among the positive regulators, we focus on P53. P53 represses the expression of SLC7A11, a component of System Xc, and also prevents the binding and activation of the NADPH oxidase NOX1 by binding to dipeptidyl peptidase-4 (DPP4), leading to ferroptosis (Jiang et al., 2015), which is associated with PD. P53 can enhance PTEN transcription by binding to its promoter. Meanwhile, PTEN can inhibit the degrading effect of Mdm2 on P53 by inhibiting the PI3K/AKT pathway, and can also enhance the stability of P53 by directly binding to it (Freeman et al., 2003). Among the negative regulators, the SNAIL factor can compete with P53 for binding to the PTEN promoter (Guo et al., 2011). In the PAM pathway, in addition to the classical PI3K/AKT/mTOR signaling pathway, the binding of P53 to PI(4,5)P2 and PI(3,4,5)P3 activates downstream Akt. By contrast, PTEN dephosphorylates PIP3 in the PAM pathway, thereby inhibiting the entire pathway (Chen et al., 2022). It is hypothesized that both P53 and PTEN can affect PD, although their regulatory mechanisms require further exploration.

Several studies have explored transcriptional regulators upstream and downstream of the PTEN signaling axis by inducing PTEN loss, although most of these studies have focused on cancer (Yang et al., 2023). However, such studies have helped to shed light on PTEN and the onset of PD, and most of these studies have investigated the mitochondrial autophagy mechanism. One study reported that XBP1s, a transcription factor that is activated in response to endoplasmic reticulum stress, transactivates PINK1, which triggers endogenous mitochondrial autophagy and thus reduces the occurrence of PD (El Manaa et al., 2021).

### 4.4 Chemical modification of PTEN

In order to function in cells, post-transcriptional PTEN needs to be accurately translated and modified. PTEN is finely regulated by a number of post-translational modifications (PTMs), and while an aberration in this regulation may lead to PTEN inactivation, it also provides an entry point for the study of functional enhancement and reactivation of PTEN (Lee et al., 2018). In addition to phosphorylation and ubiquitination, PTEN has other chemical modifications at the translational level, including SUMOylation, ubiquitination-like, acetylation, oxidation, methylation, and dimerization, to name a few, all of which subliminally regulate the function of PTEN and its role in the PAM signaling pathway (Worby and Dixon, 2014). The mechanisms of PTMs in tumors have been studied more, and mostly revolve around PTEN-induced PINK1 (Ding et al., 2019), but there are fewer related reports in PD. Nevertheless, the activation of PTEN by PTMs can trigger the activation of related kinases. One study found that PTEN can act as an upstream activator of Fyn kinase, leading to the death of dopaminergic neurons and activation of neuroinflammatory pathways, and suggested that PTEN may be a therapeutic target for PD using pharmacological inhibitors of PTEN (Johnson et al., 2023).

### 4.5 Variant subtypes of PTEN

If PTEN is mutated, even if PTEN is transcribed and translated correctly, it directly alters the physiological role of PTEN. Regarding PTEN isoforms, studies have identified translational variants of PTEN, including PTENα (PTEN-Long) and PTENβ.

PTENα is translated from an alternative start site upstream of the ATG start sequence and exhibits an addition of 173 amino acids to the standard PTEN. PTENα is a permeable membranous variant and is therefore capable of being secreted from neurons and absorbed by other cells (Putz et al., 2012; Hopkins et al., 2013). PTENα can localize in mitochondria and interact with PTEN, thereby increasing the protein level of PINK1, which regulates mitochondrial function and energy production (Liang et al., 2014). As a protein phosphatase, it allows for the dephosphorylation of phosphorylated ubiquitin (pSer 65-Ub), decreasing its levels. This process counteracts PINK1-mediated ubiquitin phosphorylation, ultimately leading to the blockade of mitochondrial autophagy (Wang et al., 2018). As mitochondrial autophagy has become an important mechanism in the pathogenesis of PD in recent years, using PTENα may be a novel strategy to inhibit PD pathogenesis.

PTENβ is another PTEN isoform that extends at the N-terminus and is manifested by the addition of 148 amino acids to standard PTEN. PTENβ is localized primarily in the nucleolus, where it dephosphorylates nucleolin, thereby negatively regulating the transcription of ribosomal DNA and biotin (Liang et al., 2017). Recent studies have found that the function of ribosomes deteriorates with age, and misfolded protein clumps trigger a “snowball effect” that leads to neurodegenerative diseases, resulting in the development of AD and PD. The reason for this may be the increased load of defective proteins, which disables the protective mechanisms that prevent protein aggregation, providing a new perspective on the study of PTENβ and PD (Stein et al., 2022).

### 4.6 PTEN and exosomes

In addition to individual cells, the transport and release of PTEN by exosomes are essential for cell-to-cell communication. Exosomes, small membrane vesicles (30–150 nm) containing complex RNA and proteins, now specifically referred to as discoidal vesicles of 40–100 nm, are the smallest type of extracellular vesicles and were first identified in sheep reticulocytes in 1983. In recent years, exosomes have received increased attention from researchers because of their potential role in the diagnosis and treatment of diseases (Kenkhuis et al., 2023).

Exosomes are involved in the pathogenesis of PD. Using autopsies of patients with PD, Braak et al. (2003) found that the distribution of α-syn can be shifted from one cell to another. This process can be mediated by several mechanisms of cellular release and uptake, among which translocation by exosomes is an important one (Valdinocci et al., 2017). Different cell types in the human brain can release exosomes, including neurons, astrocytes, and microglia. It has been demonstrated that neurons overexpressing α-syn can release and transfer α-syn to other normal neurons via exosomes (Alvarez-Erviti et al., 2011). Astrocyte-derived exosomes have also been shown to induce protein aggregation in the mouse brain (Dinkins et al., 2014). Recently, it was found that CD11 + exosomes isolated from the CSF of patients with PD contained α-syn *in vivo* and that exosomes in microglia were able to induce nigrostriatal degeneration and promote the delivery of α-syn in PD cells (Guo et al., 2020). Taken together, these findings show that exosomes function as a “transit station” in PD, and if their transporter function can be inhibited, it may be possible to indirectly prevent the progression of PD.

Phosphatase and tension homolog is also closely linked to exosomes and can be jointly involved in the regulation of PD. Recent evidence has shown that PTEN is ubiquitinated by Ndfip1 and NEDD4 at the lysine 13 site, which enables it to be secreted in exosomes and taken up by other cells (Kim et al., 2018). PTEN can also be secreted from exosomes to exert extracellular phosphatase activity (Ge et al., 2020), promoting lysosomal biogenesis and acidification. For example, it has been found that PTEN dephosphorylates transcription factor EB (TFEB) at Ser211, and defects in PTEN lead to decreased lysosomal-mediated degradation of multivesicular vesicles and increased secretion of exosomes, which promote the proliferation and invasion of cholangiocarcinoma (CCA) (Jiang et al., 2023). However, some studies on TBI have found that TBI may better activate the PAM pathway and induce ferroptosis, thereby worsening PD, with exosomes playing a role similar to the one they play in CCA. TBI can induce ferroptosis, as evidenced by increased ACSL4 expression and decreased expression of GPX4 (Wang D. et al., 2022), and this process can be reversed by exocytosis. However, exosomes can further promote the expression of PINK1 and Parkin after the occurrence of TBI (Zhang et al., 2023), which mediates ubiquitination, drives mitochondrial autophagy, and accomplishes the protective effect on PD neuronal cells (Goiran et al., 2022). Whether exosomes promote PD pathogenesis or inhibit PD progression remains to be further explored.

## 5 Discussion

Based on all of the important mechanisms involved in the regulation of PD by PTEN summarized in this review, PTEN-targeted therapeutic options for the treatment of PD have been proposed, including the development of PTEN-related drugs, gene therapy, and other innovative approaches, especially when exploring PTEN in greater depth and revealing novel PTEN-associated pathways and interactions.

Parkinson’s disease, a refractory neurodegenerative disease, is currently treated clinically with drugs, most of which aim to achieve therapeutic effects by inducing neuronal synthesis of DA, agonizing dopamine receptors, or blocking DA degradation (Jankovic, 2019). The majority of drugs aim to induce DA synthesis by neurons, agonize the dopamine receptor, or block DA degradation; such drugs include levodopa (L-DOPA), amantadine, monoamine oxidase inhibitors (MAOIs), and catechol-oxygen-methyltransferase (COMT) inhibitors. PTEN, as an oncogene, is a major player in oncology and cancer research. The current classical approach to regulate the level of PTEN is to focus on the PI3K/AKT/mTOR signaling pathway, and numerous studies have found that PTEN mutation in the PI3K/AKT/mTOR pathway can drive cancer development. Therefore, reversing the inactivation of PTEN and increasing its level of PTEN may be a more feasible approach.

Contrasting this idea of activating PTEN to inhibit the PAM pathway and thus inhibit cancer development, it may be possible to intervene in the levels of PTEN in PAM to achieve protection against PD. Since the PI3K/AKT/mTOR pathway has been shown to be protective of neurons, if we focus on the PAM signaling pathway and identify corresponding activators from upstream and downstream of PTEN, or find inhibitors or mutants of PTEN, it may be possible to activate the PAM signaling pathway, thus achieving a protective effect on PD. PTEN mutation-targeting drugs currently in clinical trials include VOX101, and the small molecule UCL-TRO-1938, a PI3K activator upstream of PTEN, has recently been found to promote neural regeneration in neurons after a stroke or some ischemia-reperfusion injury (Gong et al., 2023). Downstream mTOR activators include MHY1485 and 3BDO, among others (Wei et al., 2024). Moreover, in recent years, PTEN inhibitors have been frequently used to study PTEN-associated signaling pathways, including bpV (HOpic/bipy/pic), CAS 722494-26-0, VO-OHpic, SF1126, CAS, and curcumin (Zhang et al., 2019).

However, there remain some challenges that must be faced when developing and applying drugs. First, it is important to consider whether activation of the PAM pathway while downregulating PTEN levels also increases the risk of cancer in patients undergoing PD, which has been preliminarily explored (Mukherjee et al., 2021). Secondly, as PTEN-related drugs have not yet been applied in the clinical treatment of PD, their mode of administration and dosage are not specified, and their degree of toxicity has not been quantified or predicted. Third, the cost of these drugs may not meet public expectations. To overcome these potential difficulties, we need to broadly address the needs of patients with PD, develop more accurate drug screening platforms, and adopt rigorous clinical trials. For example, many studies have been conducted to screen small molecule drugs related to PTEN using artificial intelligence or training machine learning (Nam et al., 2023; Moskal and McQuibban, 2024). Similarly, if PTEN can be used as a “springboard” to develop related drugs, it may be possible to better serve patients with PD in the clinic, including early prevention for those with a family history of PD or slowing progression and improving prognosis for those who already have early-stage PD.

In addition to basic drug therapy, gene therapy has gradually come into the public’s view in recent years. Gene therapy studies typically use shRNAs or their cleaved siRNAs to form silencing complexes that specifically target and degrade PTEN for gene silencing. One study developed a spatially precise genetic engineering method that can monitor neuronal activity following PTEN downregulation in PD by knocking down PTEN in specific cell populations at the electrode-tissue interface via shRNA mediation (Xu et al., 2024). Many studies have promoted neuronal axon regeneration by developing new material scaffolds to piggyback siRNAs thus accomplishing a silencing effect on PTEN (Guo et al., 2019; Zuidema et al., 2020; Gao et al., 2024). Moreover, numerous studies have used innovative techniques such as bioinformatics (Ai et al., 2022), 3D spatial genomics technology (Yuan et al., 2022), and genome-wide association studies (Soutar et al., 2022) to explore the frequency of PTEN or associated genes in PD. On this basis, single-cell sequencing (Novak et al., 2022; Wasner et al., 2022) and high-throughput sequencing (Lin et al., 2019; Lechner et al., 2021) have mostly been used to explore the process of neural differentiation in PINK1-mutant cells, leading to the discovery of novel signaling pathways or associated genes and proteins that overlay PTEN (Diez-Fairen et al., 2018; Kelm-Nelson and Gammie, 2020). In addition, in recent years, CRISPR/Cas9 genome editing technology has been gradually applied to study kinase-encoding genes involved in the pathogenesis of PD, including PINK1 and Parkin, although there may be off-target effects (Mansour and El-Khatib, 2023). Some studies have established monkey models of PD at different ages using CRISPR/Cas9 and found that the phosphorylation of Parkin is important for neuroprotection in PD; similarly, CRISPR/Cas9 could be used to study PTEN in PD in future research (Han et al., 2024).

## 6 Conclusion

In conclusion, in this review, we have elaborated on the possible regulatory mechanisms of PTEN in PD in terms of phosphorylation, ubiquitination, mitophagy, RNA molecules, exosomes, and many other aspects, thus refining the pathogenesis of PD (Table 1). Although we have revealed the strong correlation between PTEN and PD, most of the abovementioned mechanisms have not been fully elucidated, especially how PTEN regulates the balance of neuronal cell proliferation in patients with PD and thus exerts neuroprotective effects. Hence, a genetic database of associations between PD and PTEN must be established to improve the prediction ability of PD. More advanced assays need to be developed to monitor PTEN expression levels in different patients with PD, and PTEN-related therapeutic techniques need to be applied in the clinical treatment of PD. It is believed that in the future, further basic research will lead to the application of PTEN in the clinical treatment of PD, thereby bringing benefits to the majority of patients.

**TABLE 1:** Effect of different substances in the regulation of phosphatase and tension homolog (PTEN) in Parkinson’s disease (PD).

Mode of the regulation	Name of the material	Role of the substance in PD	Mechanism	References
Phosphorylation	PTEN	Promote	Dephosphorylates PIP3 to PIP2 as a lipid phosphatase, preventing Akt activation by PI3K	Masson et al., 2016
Ubiquitination	Ndfip1	Inhibit	Mediates PTEN entry into the nucleus and deregulates AKT inhibition	Goh et al., 2014
			Control of nuclear region compartmentalization of PTEN regulates cell proliferation	Howitt et al., 2015
	NEDD4	Inhibit	Ubiquitination of PTEN degrades it and promotes neurite growth	Christie et al., 2012
			PTEN promotes neuronal survival by upregulating its levels via CAMP/PKA	Ahn et al., 2008
Ferroptosis	GCLM	Inhibit	PTEN deletion upregulates GCLM expression levels, promotes GSH synthesis and inhibits iron death	Kezhuo et al., 2022
RNA	miR-221	Inhibit	Inhibition of 6-OHDA-induced PC12cell autophagy by PTEN/Akt/mTOR	Zhou et al., 2021
			Directly targets PTEN, and PTEN over-expression eliminates the protective action of miR-221 on p-AKT expression in PC12 cells	Zamanian et al., 2024
	miR-299-5p	Inhibit	Targeting SP3 inhibits apoptosis in a PD cell model with the aid of the PTEN/AKT/mTOR pathway	Yejun et al., 2023
	miR-410	Inhibit	Neuroprotective effects on 6-ohDA-induced PD cell models by modulating the PTEN/AKT/mTOR signaling axis	Ge et al., 2019
	miR-381	Inhibit	Binding to EGR1 upregulates PTEN/P53 to alleviate PD	Guo et al., 2023
	miR-181b	Inhibit	Inhibition of cellular autophagy via PTEN/Akt/mTOR reduces cellular damage in PD	Li et al., 2018
	miR-27a/b	Inhibit	Inhibition of PINK1 translation inhibits autophagic clearance of mitochondria by lysosomes and reduces PD	Kim et al., 2016
	miR-1976	Promote	Overexpression of miR-1976 targets PINK1 to increase apoptosis and mitochondrial damage in dopaminergic neurons, thereby increasing the risk of PD	Qiu et al., 2023
	miR-96	Inhibit	PPX attenuates MPP + -induced cellular and MPTP-induced neuronal damage in mice by directly reducing miR-96 activation of bnip3-mediated mitochondrial autophagy	Wang et al., 2021
	circEPS15	Inhibit	Rescue of DA neuronal degeneration by miR24-3p/PINK1 axis-mediated improvement of mitochondrial function	Zhou et al., 2023
	miR-873	Unknown	Direct targeting of Ndfip, indirect effects on PTEN/Akt pathway and ubiquitination of PTEN upon binding to NEDD4	Howitt et al., 2012, Howitt et al., 2015
	LncRNASNHG1	Promote	Targeting miR-153-3p stimulates PTEN/Akt/mTOR signaling and induces SH-SY5Ycell neurotoxicity	Zhao J. et al., 2020
	LncRNA TUG1	Promote	Modulation of miR-152-3p/PTEN pathway to promote PD	Zhai et al., 2020
	GAS5	Promote	Binding to miR-150 targets Fosl1, activating the PTEN/AKT/mTOR pathway, which promotes apoptosis, inhibits neuronal activity, and accelerates PD progression	Ma et al., 2022
	SOS1-IT1	Inhibit	Reduce damage to MPP + -induced SK-N-SH cells by miR-124-3p, which is reversed by overexpression of PTEN, a target of miR-124-3p	Fan et al., 2022
Exosome	Exosome	Unknown	PTEN is ubiquitinated by Ndfip and NEDD4 and secreted in exosomes, where it is taken up by the cell or secreted outside the cell to exert phosphatase activity	Kim et al., 2018, Ge et al., 2020
Transcription	P53	Promote	Enhancement of its transcription and stability by binding to the PTEN promoter	Freeman et al., 2003
			PTEN inhibits Mdm2 degradation of P53 by inhibiting the PI3K/AKT pathway	Freeman et al., 2003
	SNAIL	Promote	Competition with P53 for binding to the PTEN promoter	Guo et al., 2011
Variation and modification	PTENα	Inhibit	Dephosphorylates the phosphorylated ubiquitin pSer65-Ub, leading to a decrease in its level, counteracting PINK1-mediated ubiquitin phosphorylation and blocking mitochondrial autophagy	Wang et al., 2018
	PTENβ	Promote	Dephosphorylation of riboprobes to negatively regulate transcription of ribosomal DNA and ribosomal biotin	Liang et al., 2017
